# Heterogeneity among Clinical Intestinal Escherichia coli Isolates upon Acquired Streptomycin Resistance

**DOI:** 10.1128/spectrum.03500-22

**Published:** 2023-05-15

**Authors:** Lacey R. Lopez, Claire M. Miller, Joanna N. Jeyachandran, Chuang Li, Kenneth W. Simpson, Janelle C. Arthur

**Affiliations:** a Department of Microbiology and Immunology, University of North Carolina at Chapel Hill, Chapel Hill, North Carolina, USA; b Department of Clinical Sciences, College of Veterinary Medicine, Cornell University, Ithaca, New York, USA; c Center for Gastrointestinal Biology and Disease, University of North Carolina at Chapel Hill, Chapel Hill, North Carolina, USA; d Lineberger Comprehensive Cancer Center, University of North Carolina at Chapel Hill, Chapel Hill, North Carolina, USA; Agriculture and Agri-Food Canada

**Keywords:** *Escherichia coli*, adherent-invasive, aggregation, clinical strains, motility, rpsL, streptomycin

## Abstract

Escherichia coli isolates from inflammatory bowel disease (IBD) patients are often multidrug resistant, including to streptomycin. Streptomycin resistance (Str^R^) mutations can alter bacterial behavior, which may influence intestinal disease. We generated a spontaneous Str^R^ strain of the intestinal adherent-invasive E. coli (AIEC) strain NC101. Whole-genome sequencing revealed a single missense mutation in *rpsL* that commonly confers Str^R^, *rpsL*-K43N. Str^R^ NC101 exhibited a striking loss of aggregation and significantly increased motility, behaviors that can impact host-microbe interactions. Behavioral changes were associated with reduced transcription of *csgA*, encoding the biofilm component curli, and increased transcription of *fliC*, encoding flagellin. Scanning electron microscopy (SEM) detailed morphologic changes consistent with the observed alterations in multicellular behavior. Because intestinal E. coli isolates exhibit remarkable strain-specific differences, we generated spontaneous Str^R^ mutants of 10 clinical E. coli phylotype B2 strains from patients with IBD, colorectal cancer, and urinary tract infection. Out of these 10 Str^R^ clinical strains, two had altered colony morphology on Congo red agar (suggesting changes in extracellular products), and three had significant changes in motility. These changes were not associated with a particular *rpsL* mutation nor with the presence of virulence genes encoding the inflammation-associated E. coli metabolites yersiniabactin or colibactin. We conclude that common mutations in *rpsL*, which confer Str^R^, can differentially alter disease-associated phenotypes across intestinal E. coli strains. These findings highlight the heterogeneity among seemingly similar intestinal E. coli strains and reveal the need to carefully study the strain-specific effects of antibiotic resistance mutations, particularly when using these mutations during strain selection studies.

**IMPORTANCE** We demonstrate that Str^R^, commonly acquired through a single point mutation in *rpsL* (a gene encoding part of the 30S bacterial ribosome), strikingly alters the morphology and behavior of a key intestinal AIEC strain, NC101. These changes include remarkably diminished aggregation and significantly increased motility, traits that are linked to AIEC-defining features and disease development. Phenotypic changes were heterogeneous among other Str^R^ clinical E. coli strains, underscoring the need to evaluate the strain-specific effects of commonly acquired antibiotic resistance mutations. This is important, as the results of studies using mutant Str^R^ *Enterobacteriaceae* strains (e.g., for cloning or *in vivo* selection) may be confounded beyond our demonstrated effects. Long term, these findings can help researchers better distinguish the contribution of specific E. coli traits to functional changes in the microbiota. Evaluating these strain-level differences could provide insight into the diversity of IBD symptoms and lead to improved therapies for microbiota-driven intestinal disorders.

## OBSERVATION

Escherichia coli isolates from IBD patients are frequently multidrug resistant, especially to aminoglycosides like streptomycin (1). Streptomycin resistance (Str^R^) can be acquired through spontaneous mutations in *rpsL*, which encodes the 30S ribosomal protein S12 (2). Str^R^ is frequently caused by a missense mutation in *rpsL* that results in the substitution of lysine with asparagine at codon 42 or 43 (3). In this study, we reveal that the acquisition of Str^R^ induced distinct behavioral and morphological changes in intestinal adherent-invasive E. coli (AIEC) strain NC101, similar to those observed in related pathogens (4–7). However, these alterations were not consistent among 10 additional, predominantly intestinal, clinical E. coli strains from the same B2 phylogroup as NC101. Inducing Str^R^ is a common laboratory approach used during strain selection, and our results demonstrate that mutations in *rpsL* may not be as neutral as some may assume. Additionally, our studies highlight the importance of examining strain-specific behavioral traits that may alter host-microbe interactions with intestinal E. coli.

### The *rpsL*-K43N Str^R^ mutation drives behavioral and morphologic alterations in AIEC NC101.

**FIG 1:**
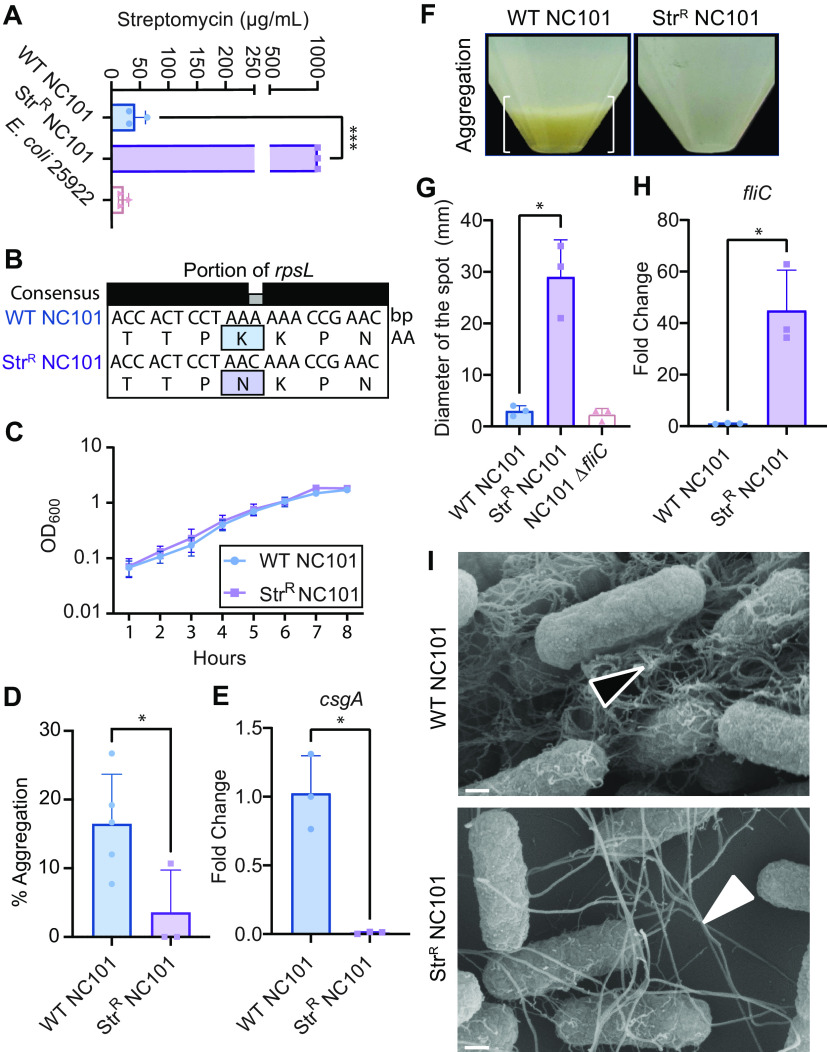
(A) MIC assays confirmed Str^R^. (B) *rpsL* gene sequencing. (C) Growth curve in MM. (D) Aggregation quantification of panel F. (E) *csgA* expression, normalized to *uidA*, expressed as the fold change to WT NC101. (F) Aggregation (white brackets). (G) Motility swarms in MM. (H) *fliC* expression. (I) SEM (×100,000; scale bar, 200 nm); the open triangle points to curlinacious fibers, and the closed triangle points to flagella-like products. All graphs represent the means ± standard error of the mean. *, *P* < 0.05; ***, *P* < 0.001; Welch’s *t* test.

E. coli NC101 is a proinflammatory and procarcinogenic intestinal AIEC strain (6–9). To examine how Str^R^ impacts the function of this broadly used strain, we generated a spontaneous Str^R^ NC101 strain by passaging wild-type (WT) NC101 with increasing amounts of streptomycin and validated resistance via an MIC assay (Fig. 1A). Whole-genome sequencing revealed that Str^R^ NC101 had only a single missense mutation in codon 43 of the *rpsL* gene, resulting in the substitution of lysine (K) with asparagine (N). We termed this mutation *rpsL*-K43N (Fig. 1B). Although there was no difference in the growth of WT and Str^R^ NC101 cells in minimal defined medium (MM) (10) (Fig. 1C), Str^R^ NC101 cells failed to aggregate in liquid culture, suggesting changes in extracellular product production (Fig. 1D and F).

As the extracellular product curli is an important component of *Enterobacteriaceae* biofilms, we assessed the expression of the curli biosynthesis gene *csgA*. Indeed, Str^R^ NC101 expressed significantly less *csgA* (Fig. 1E). Because aggregation and swimming motility are often inversely activated, and altered motility can be a feature of AIEC (11), we measured the bacterial motility. As expected, Str^R^ NC101 had significantly increased motility versus WT NC101 and the nonmotile control strain NC101 Δ*fliC* (Fig. 1G). The motility changes matched an upregulation of the flagellar biosynthesis gene *fliC* in Str^R^ NC101 (Fig. 1H). Scanning electron microscopy (SEM) revealed changes to the cellular morphology between WT and Str^R^ E. coli NC101, where WT cells had an abundance of fibers resembling curli and Str^R^ NC101 predominately had flagella-appearing structures (11–13) (Fig. 1I). These data suggest that the *rpsL*-K43N mutation in NC101 confers Str^R^ and, in MM, mediates a switch from the sessile to motile state for this important AIEC strain.

### Acquired Str^R^ has differential effects across clinical E. coli strains.

**FIG 2:**
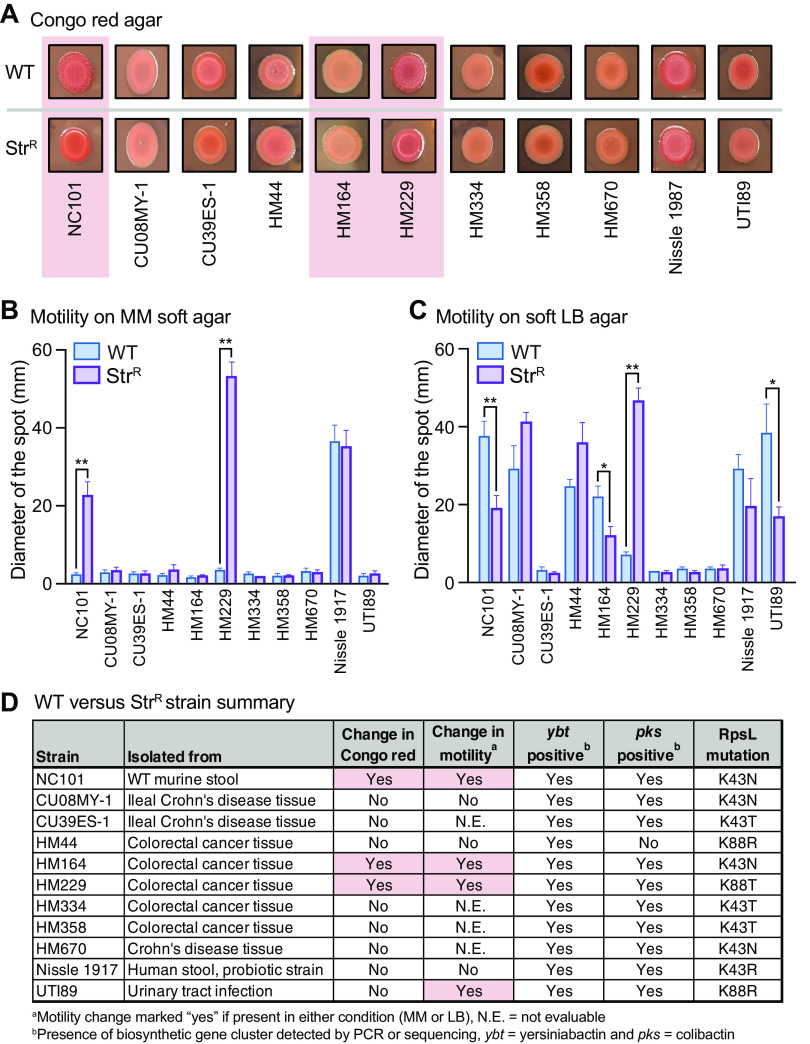
(A) Congo red morphology; the pink boxes indicate strains with phenotypic changes. (B and C) Motility swarms on MM (B) and LB (C) (mean ± standard error of the mean; *, *P* < 0.05; **, *P* < 0.01; *t* test, *n* = 3 to 7). (D) Strain table showing the phenotypes, virulence genes, and *rpsL* mutations; the pink boxes indicate key changes in WT versus Str^R^ strains.

E. coli isolates exhibit remarkable strain-specific differences in genome composition, behavior, and disease-inducing potential. Thus, we generated Str^R^ isolates of 10 clinical E. coli strains, predominantly isolated from intestines and of the same B2 phylotype as NC101. None of these strains aggregated in MM; therefore, to qualitatively determine if Str^R^ was associated with broad changes in extracellular products, we plated the strains on Congo red agar. WT NC101 displayed a red, dry, and rough (RDAR) morphology, previously shown to be dependent upon the production of curli and cellulose (14, 15). In contrast, Str^R^ NC101 presented as red and smooth on Congo red, supporting observations in Fig. 1. Congo red also revealed that two strains, HM164 and HM229 (16), had similar changes as NC101 upon acquiring Str^R^ (Fig. 2A).

We next performed motility assays on MM and LB soft agar, as nutrient availability can alter bacterial motility (17). Indeed, the motility of seven strains appeared different between MM and LB (Fig. 2B and C). More importantly, Str^R^ significantly altered the motility of NC101 and three clinical strains (HM164, HM229, and UTI89), three were unaffected (CU08MY-1, HM44, and Nissle 1917), and the remaining four were nonmotile in the tested conditions (Fig. 1G and 2B to D).

Since there are several mutations in *rpsL* known to confer Str^R^, we sequenced the *rpsL* gene of each WT and Str^R^ strain. Sequencing revealed various *rpsL* mutations (Fig. 2D), suggesting that the observed changes in Congo red morphology and motility were not driven by a particular *rpsL* mutation. Furthermore, these mutations were not associated with the presence of *ybt* or *pks* genes, which encode disease-modulating metabolites yersiniabactin and colibactin (6, 8) (Fig. 2D). Taken together, we conclude that the effects of Str^R^ on E. coli morphology and behavior are heterogeneous and driven by strain-specific differences.

In summary, we reveal that the common *rpsL*-K43N mutation conferring Str^R^ is associated with dramatic changes in AIEC NC101 behavior, notably the production of extracellular products and motility. Extracellular products and motility impact proinflammatory behaviors, and hypermotile lineages of E. coli can be used to distinguish Crohn’s disease patients from healthy controls (11, 15, 16, 18, 19). However, these morphological changes were inconsistent among NC101 and the 10 Str^R^ clinical E. coli strains. These findings highlight the importance of evaluating phenotypic changes across multiple E. coli strains that inhabit the intestinal niche, rather than generalizing conclusions based on the results from one strain, particularly in studies using antibiotic resistance mutations (11, 18, 20–22).

With the broad usage of antibiotics, there is a surge of antibiotic resistance across resident intestinal bacteria (1, 23, 24). One study found that an increased proportion of *Enterobacteriaceae* isolates from IBD patients were ciprofloxacin and rifaximin/rifampicin resistant versus isolates from healthy individuals (1). In addition, similar to our study, changes in aggregation/motility have been associated with fluoroquinolone resistance and can enhance AIEC-defining characteristics (9, 11, 15, 25). Thus, it is important to evaluate disease-associated E. coli traits in response to these and other clinically important antibiotics. These findings may help explain the broad functional variation among genetically similar gut microbiota members and better inform treatment for IBD-related disorders.

### Methods.

**(i) Bacterial strains.** The NC101 and NC101 Str^R^ sequences are available at GenBank under accession numbers CP072787 and CP070227; the strains were generated as previously described (21). Strains CU39ES-1 and CU08MY-1 were from Kenneth W. Simpson at Cornell University (22); strains HM44, HM164, HM229, HM334, HM358, and HM670 were a gift from Barry Campbell at the University of Liverpool (16); and NC101 (6, 7), ATCC 25922 (26), and Nissle 1917 (26) were archived in the Arthur lab at the University of North Carolina (UNC), Chapel Hill. UTI89 was from Harry Mobley at the University of Michigan Medical School, and NC101 Δ*fliC* was from Melissa Ellermann at the University of South Carolina, Columbia.

**(ii) Media and growth conditions.** The strains were stored at −80°C with glycerol, and E. coli cells were grown at 37°C with shaking at 220 rpm in MM (containing nicotinic acid, as NC101 is an auxotroph) (10). The aggregation and Congo red assays were previously described (15). For the motility assay, MM or LB soft agar plates (0.25% [wt/vol] agar) were inoculated with 1 μL overnight culture, and the motility swarm diameters were measured after 6 h (LB) or 8 h (MM) at 37°C. For the MIC assay, streptomycin was diluted 2-fold from 1,000 μg/mL in 200 μL/well MM and inoculated with 2 μL overnight bacterial culture. After 12 to 18 h at 37°C with shaking at 220 rpm, the MIC was recorded as the lowest concentration of streptomycin required to prevent E. coli growth (optical density at 600 nm [OD_600_]). ATCC 25922 was a susceptible control strain.

**(iii) Transcriptional analysis.** Bacterial overnight cultures were back-diluted to an OD_600_ value of 0.05 in MM and grown at 37°C with shaking at 220 rpm for 8 h. Quantitative PCR (qPCR) was performed as previously described (26). Threshold cycle (*C_T_*) values were normalized to *uidA.* The primers used were as follows: *uidA* forward (*uidA*-F), 5′-TCAACAGACGCGTGGTTACAGTCT-3′; *uidA* reverse (*uidA*-R), 5′-TCCATCGCAGCGTAATGCTCTACA-3′; *csgA-*F, 5′-GGTAATAACAGCGGCCCGAA-3′; *csgA-*R, 5′-TGTCATCAGAACCTTGGCCC-3′; *fliC-*F, 5′-ACAACTACTGAGGATGCGGC-3′; and *fliC-*R, 5′-GCGGAATTTGTCGATCTGGC-3′.

**(iv) SEM.** SEM was performed at the Microscopy Services Lab (MSL) at UNC on bacteria pelleted from 8-h cultures in MM. The samples were imaged using a Zeiss Supra 25 FESEM device (5 kV, SE2 detector, 5 mm working distance, 30-μm aperture; Carl Zeiss SMT Inc., Peabody, MA).

**(v) Targeted *rpsL* sequencing.** A region of *rpsL* encoding amino acids 30 to 98 was amplified for Sanger sequencing (Genewiz/Azenta Life Sciences): F, 5′-AAACGTGGCGTATGTACTCG-3′, and R, 5′-TTGGAACGAGCCTGCTTAC-3′.

**(vi) Rigor and reproducibility.** All data are representative of at least three independent experiments.
